# Uterine Myomata1Read at the 36th annual meeting of the Medical Association of Central New York, held at Auburn, September 22, 1903.

**Published:** 1904-04

**Authors:** A. L. Beahan

**Affiliations:** Canandaigua, N. Y.


### Uterine Myomata.[1]

By A. L. BEAHAN, M. D., Canandaigua, N. Y.

UTERINE fibroids, properly speaking uterine myomas, have been of frequent occurrence during the past forty years. Though not found in native Africans, they are more common in American negro women than in the whites of any country. They occur as soft, rapid growing cystic tumors, uniform in contour, often misleading and simulating pregnancy, as multinodular hard masses, readily shelled out of inadherent capsules, or as polypi extrude from the uterus or remain in the uterine or cervical canal, being attached by a fibrous pedicle to the wall of the uterus. The soft variety develops at any age, and Tait found it was not influenced by removal of uterine appendages. The hard multinodular variety on the contrary, is associated with menstrual disturbances, as dysmenorrhea and menorrhagia, and is intimately related to the menstrual life. This is so true that if the menstruation is prolonged, suspicions are at once directed toward

1. Read at the 36th annual meeting of the Medical Association of Central New York, held at Auburn, September 22, 1903.

uterine myoma as a probable cause. Hemorrhage from this overtime menstrual life, probably irregular, always excessive, causing anemia and its sequences are the ends attained by these growths. These are classical, undisputed facts. We are not much wiser about this kind of a tumor than we were two or three decades ago, that is, as to its cause or its anatomy. There is but little need of reciting facts well appreciated, if not altogether understood.

How to diagnosticate uterine myomas is important. The rapidly growing soft tumors, occurring as they frequently do during menstrual life, are the most difficult. They simulate in many ways pregnancy. Dilating the uterus and exploring is, of course, undesirable. There is no operator of experience, perhaps, but has been mistaken except possibly by the open incision abdominal diagnosis. Even this has not proven a sure way and the pregnant uterus has been incised before the error has been discovered. The hardened cervix, irregular uterus, slow growth of the tumor, and severe hemorrhages, make the multinodular myomas easier to diagnosticate. Dilatation as a means for determining the presence of uterine growths is dangerous, as is the use of the uterine sound. Sloughing of the soft myomas is likely to result and in the hard forms the capsule may be perforated and the low vitalised tissue be harmed. If the appendages be adherent to the uterus, peritonitis may ensue. Between the dangers of sloughing of the growth and the consequent sepsis and inflammatory developments, life is imperiled and nothing gained that could not have been compassed by as safe means. The educated finger touch can discern more than the conjoined instrument and finger, or instruments alone. A bivalve speculum and a probe or sound or a Sims speculum with tenaculum to draw cervix well down and dilator to open the cervical canal are diagnostic techniques for determining diagnosis of uterine myomas, crude, unscientific, vicious.

Given the presence of uterine myomas, what shall be advised? Two salient features, hemorrhage and pain, must be appreciated. Anemia, exsanguination, impaired vital resistance, neurasthenia, mental depression and irritability mean a useless invalid life in which any accidental disease may kill. Pain due to pressure, to ulceration, softening and septic changes, or the extrusion of a pedunculated mass is significant but not so readily sifted in its bearings on the question at stake. The rapidly growing, soft myoma must be removed. No medicine, no tentative procedure is justifiable. The removal of the appendages will not stop the development of the growth, but the serous secreting membranes lining the sacculated interior and the soft mushy contents increase and proliferate unceasingly and uncontrollably.

The hard myomas, developing slowly, insidiously, if on the peritoneal surface cause at the most a local peritonitis; if under the uterine mucosa they give early only a modification of menstrual flux and disturbance, suggestive, but not pathologic, concealed and unmentioned among the category of woman's secrets. There are two extremes of views held by equally honest reasoners about hard uterine tumors. One which believes in early interference and contends that the dangers of fibroid polypi, degenerative changes, tendency to sarcomatous changes, pressure ulceration of surface peritonitis, or a low grade of peritonitis from tumor presence, concommitant disease of the appendages and the general constitutional disturbances, making toward chronic invalidism, warrant in the wealthy classes and demand in the toilers and wage-earning women, operative interference quite early in the major manifestations of this disease. The other extreme of view regards medicinal measures of expectant character, that is symptomatic treatment to be demanded. Ergot, cannabis Indica, hydrastis, aromatic sulphuric acid and gallic acid, represent the hemostatic drugs. Hot vaginal douches, rest in bed and some form of electricity or cold applied to spine are useful expedients. Uterine tinkering by means of specula and medicated swabs are commercial methods of treating uterine myoma.

The tonic treatment where building up becomes necessary must be by limiting the hemorrhages and supplying new blood. Rest in bed and hot douches with moderate medication is advised. The use of iron is of doubtful propriety, especially in heroic doses, but normal saline rectal enemas, care of the skin and other functions, with a general mixed diet and moderate amount of light alcoholic stimulants will be found beneficial. Too much officiousness on the part of the physician is a nuisance. The patient, from the nature of the disease, has a self-centered state of mind. True help must negative, not accentuate, this morbidity. Frequent vaginal examinations and speculum applications are never needed and are not to be commended. The reason becomes emphatically obvious why uterine probes, sounds and curettes are dangerous when the capsule of the separate hard myomas are examined. They are often of delicate structures, sparsely supplied with bloodvessels and were it not that a small amount of blood supply suffices for their vitality, every traumatism would prove a disastrous climax.

The pathology of the uterine mucus membrane in myomatous conditions can be removed by gauze wrapped on a simple applicator. The mucosa is not intrinsically the diseased structure. It may be incidentally diseased by a fibroid polypus in its process of enucleation and expulsion. The use of curets of simple or barbarous type may have a moral effect on the patient's mind or the imagination of the operator. It may cut off a few bloodvessels and diminish temporarily that little of hemorrhagic area, but it is only then a temporising expedient and if a myoma is already being extruded, the injury inflicted to the compressed polypus will cause sloughing and sepsis. There is a justification in letting hard uterine myoma alone. There is no warrant in meddling and in this category, electricity must be placed. The question of whether a tumor is greater or smaller than at a previous examination is a large proposition to settle. The devotees of any fad can easily demonstrate to the mind of a confiding, trusting patient the improvement so much desired, and gas accumulations, retained feces, new positions of the growth, even the condition of the mind, the personal equation of the examiner, exclusive of questions of skill and experience, may add to the confusion of diagnosis in this particular form of disease.

The need for operative interference is determined by the condition of the patient's necessity for severe labor, the question of hemorrhage, pain, and complications as of sarcomatous degeneration, sloughing and ulcerative areas at points of pressure as against fecal accumulations in the rectum. The choice of operation offers a wide field. What success does this or that technic afford? The history of operative interference for uterine myoma is recent. In 1872, Tait began removing the uterine appendages to cure uterine myomas after witnessing two deaths in succession in enucleation cases,—a sad comment on the morcellation fad by this master of all gynecological minds. Considering the short history of cures of uterine growths and the brilliant plans evolved, it is no wonder that many methods have been employed. It would not be profitable to try to enumerate and describe these technics. What plan is safest for the greatest number of operators? Which gives the best results?

Statistics are not as reliable as wished for. It is necessary for us to digest all facts, observe results and form personal conclusions. In gynecological surgery, it being as stated a young science, this truth is especially patent. In our own country, with fertility of inventive genius, it is even more demanded that we sift for ourselves. Does morcellation of fibroids by the superior roüte, appeal to our judgment as wise? It is easy and one after another nodule may readily be enucleated, but are we sure that all the separate nodules are removed? If not, they will grow to the full size of such former tumors and require a secondary operation. Should morcellation be attempted from below? Never! It is bad enough to leave a flabby, bruised, oozing remnant of a uterus to hopeful phagocytosis in the abdominal cavity, but to push up through the septic vagina and violently remove a fibroid tumor or nodule, disgraces surgery.

The vaginal route is only a sentiment. It needs no weighing in true uterine myomas. If malignancy has developed, this route, because of the demand for proper drainage, is the only way to proceed, but the vaginal vault is weakened by adopting the inferior route and nothing is gained of any account. Abdominal hysterectomy for uterine myomas seems the best means for their removal. Two plans are used. The first and oldest removes the uterus and tumor at the cervix, a constricting band encircling the cervix below the body of the uterus and its myoma, the stump being placed extraperitoneally in the lower angle of the wound and held there by proper devices till the part of tissue above the constriction separates and that about the incision shuts off the abdominal cavity, granulates and becomes sound tissue.

The second method, or the dropped pedicle requires the bloodvessels to be ligated on either side, including broad ligaments, round ligament and close to cervix. The fore and aft incision with flaps, and incision through cervix with approximation of flaps over the stump of cervix, the canal of which is planned to be closed by suturing, or the incision is fore and aft just at the cervico-vaginal juncture and closure is to be by peritoneum matching peritoneum, mucous membrane joining mucous membrane. What are the dangers of the superior route and complete, clean operative work? Hemorrhage, damage to other organs, as ureters, intestines, omentum or bladder and sepsis. The discussion of these matters is relevant. Given in either case a tumor of fair size and uncomplicated, the exceptions may always require a modification toward the other technic. Hemorrhage is especially to be dreaded in this anemic disease. Abdominal hemorrhages especially, even if small in amount, cause shock out of all proportion to the amount of blood lost and exsanguination makes tardy convalescence, slow healing, and invites sepsis by small resistance to pus organisms. The constriction method, best represented in the wire serre-noeud of Köberle, loses only the blood in the excised uterus and the small amount of the external and fore and aft incision. A description of this method step by step is as follows:

1. Opening of abdomen from pubes, above through linea alba of sufficient length to deliver myomatous uterus.

2. Expressing uterus, using myoma screw to expedite process.

3. Incision fore and aft well up above uterocervical juncture, avoiding at the sides the bloodvessels of the appendages and in front the bladder's reflection on the uterus.

4. Pushing down with gauze pads these fore and aft peritoneal flaps from off the uterus.

5. Placing hemostats on the peritoneal flaps, three in front and one behind.

6. Clamp with vaginal hysterectomy clamps, one on either side perpendicular to tumor at angle of fore and aft incisions, the bloodvessels of the appendages.

7. Place wire of serre-noeud about tumor tightly, just below clamps and hemostats, which are the guides to flap.

8. Apply two more vaginal hysterectomy clamps, one on either side to the vessels of ovaries and tubes.

9. Slightly tighten noeud.

10. Apply two pins crosswise; one near anterior cervical part, but just posterior to vaginal hysterectomy clamps to include the bloodvessels of either side. The second pin is put about one-third of distance from posterior side of included tissue.

11. Tighten serre-noeud after examining to see that the anterior and posterior flaps are drawn carefully, but not with over tension, into the bite of the serre-noeud and that no bladder fold is encircled by the noeud, to give latter a vesical fistula. The noeud is then tightly drawn by means of wrapping the wire about the jaws of a vaginal hysterectomy clamp and when the wire is well tightened, the end of the wire is wrapped by turns about the handles of the noeud. The tightening is increased by the key of the serre-noeud, just sufficient for hemostasis to be safe.

12. Cut off with bistoury the tumor and uterus above pins and ligate pedicles, using pedicle needle and the strong silk ligatures used in ovariotomy.

13. Draw fore and aft flaps over stump and suture toward each other, not necessarily together, to avoid retraction.

14. Retighten serre-noeud by means of key.

15. Introduce the abdominal closing sutures through and through, silkworm gut from above down, putting a No. 1 silk suture from peritoneum of one side to the stump and then to the peritoneum of other side of the incision, having crowded the stump hard down into the lower angle of the incision against the pubes and tying the silkworm gut sutures from above down, having first tied the peritoneal to stump suture, whose purpose is the immediate shutting off of the abdominal cavity. Secure union about the stump may be expected in forty-eight hours.

16. Tie to end of pins of one side, strong silk thread. Apply the edges of folded gauze, one piece against the other, over the stump, bring the ends of the silk thread over gauze and tie to the other side on ends of pins. A flat piece of gauze doubled is carefully insinuated from without inward on either side under the pins to protect the skin from their pressure and a quantity of absorbent cotton is placed over stump and wound dressings and the many-tailed abdominal flannel woolen bandage applied.

This is the technic as practised so successfully by Dr. Joseph Price. Having personally examined many cases at the Philadelphia dispensary of years' standing, the proof is positive that no sequels of hernia of abdominal wall or vaginal outlet were found. The dropped pedicle operation, popularised by Baer, is a fore and aft double flap tying often over, hemostats on either side of cervix, first in ligaments and last close to cervix. The deep suturing about the cervix requires wide-cutting curved needles that leave over large openings not filled with the suture material. If the vagina is opened there is danger of sepsis. If the cervical canal is attempted to be closed, it cannot be done by suturing the walls of the canal together because this membrane does not unite readily and it is poor tissue to cut out a cup-shaped cavity from and then approximate because of its cartilaginous nature. It is difficult to avoid leaving a dead space under the peritoneal flaps on top of the stump. In the exsanguinated or profoundly anemic patient, this result invites sepsis, and sepsis in the depth of the pelvis under these circumstances means immediate septic peritonitis, or remote dead ligatures, abscess, adhesions and chronic invalidism. The dropped pedicle is the easier operation, but the point of possible septic infection is also dropped into the depths of the pelvis.

The recent renewal of morcellement, the catheterisation of the ureters, as a step of the operation is a quiet insinuating, persistent, unanswerable argument against the intraperitoneal method and against its safety because of danger of cutting or ligating ureters, secondary hemorrhage, dead ligatures and sepsis. Where adhesions exist, such an operation is the best, or where the tumor is very small in size and has ulcerated, a difficulty might be experienced in making a pedicle long enough to be grasped in the lips of the abdominal incision and be held by the pins and serre-noeud without undue tension. The dropped pedicle is less objectionable than the removal of the cervix, especially if vaginal gauze drainage is used, as then the vaginal vault is weakened, but the suspension of the cervix from the abdominal wall in the retroperitoneal method makes for the working woman a pelvic floor, better than senile changes give, and for any woman, makes for comfort and normal pelvic relations.

The vulnerable points of the intraabdominal and at present popular procedure have been covered. It remains to emphasise the like consensus of conclusions relating to the extraperitoneal abdominal method. The foci of infection are without, walled off by the suture that closes the gap above the stump by the encircling raw surface of peritoneum that fastens with jealous care about the cervical stump and quickly closes the opening. No hemorrhage can be expected, no dead ligature anticipated, no dead space formed. The patient is at once comfortable and safe. The color comes to the lips as the blood is rapidly renewed. The bowels move soon; the kidneys assume quickly their functions, as shock is slight and at the end of ten days the abdominal silkworm gut sutures are removed. No pus should have developed, even in the stitch holes. Nothing is left in the abdominal cavity to ever cause trouble. The serre-noeud should be tightened every twenty-four to forty-eight hours, and the external part of the stump should be healed in about fourteen days, and at the end of four to five weeks the wound is soundly healed.

There can be no more gratifying result in gynecological surgery, none more safely applied by the large body of surgeons, now doing splendid and saving surgery in the remote places of the country; none, if statistics were carefully and honestly compiled, that should meet with warmer approval if properly appreciated and skilfully and faithfully carried out; none in anemic subjects so free from danger, than the operation for uterine myomata detailed in the foregoing description.

